# Increased large conductance calcium-activated potassium (BK) channel expression accompanied by STREX variant downregulation in the developing mouse CNS

**DOI:** 10.1186/1471-213X-6-37

**Published:** 2006-07-27

**Authors:** Stephen H-F MacDonald, Peter Ruth, Hans-Guenther Knaus, Michael J Shipston

**Affiliations:** 1 Centre for Integrative Physiology, School of Biomedical Science, Hugh Robson Building, University of Edinburgh, Edinburgh, Scotland, EH8 9XD, UK; 2 Pharmacology and Toxicology, University Tuebingen, Institute of Pharmacy, 72076 Tuebingen, Germany; 3 Division for Molecular and Cellular Pharmacology, Department of Medical Genetics, Molecular and Clinical Pharmacology, Medical University Innsbruck, Peter-Mayr Strasse 1, 6020 Innsbruck, Austria; 4 Current address: Trinity Institute of Molecular Medicine, St. James's Hospital, Dublin 8, Republic of Ireland

## Abstract

**Background:**

Large conductance calcium- and voltage activated potassium (BK) channels are important determinants of neuronal excitability through effects on action potential duration, frequency and synaptic efficacy. The pore- forming subunits are encoded by a single gene, *KCNMA1*, which undergoes extensive alternative pre mRNA splicing. Different splice variants can confer distinct properties on BK channels. For example, insertion of the 58 amino acid stress-regulated exon (STREX) insert, that is conserved throughout vertebrate evolution, encodes channels with distinct calcium sensitivity and regulation by diverse signalling pathways compared to the insertless (ZERO) variant. Thus, expression of distinct splice variants may allow cells to differentially shape their electrical properties during development. However, whether differential splicing of BK channel variants occurs during development of the mammalian CNS has not been examined.

**Results:**

Using quantitative real-time polymerase chain reaction (RT-PCR) Taqman™ assays, we demonstrate that total BK channel transcripts are up regulated throughout the murine CNS during embryonic and postnatal development with regional variation in transcript levels. This upregulation is associated with a decrease in STREX variant mRNA expression and an upregulation in ZERO variant expression.

**Conclusion:**

As BK channel splice variants encode channels with distinct functional properties the switch in splicing from the STREX phenotype to ZERO phenotype during embryonic and postnatal CNS development may provide a mechanism to allow BK channels to control distinct functions at different times of mammalian brain development.

## Background

Large conductance calcium- and voltage- activated potassium (BK) channels are key determinants in the regulation of vertebrate neuronal excitability by controlling action potential duration, firing frequency, spike frequency adaptation and neurotransmission [1-5]. In the adult vertebrate nervous system BK channels are widely expressed and are located in both pre- and post- synaptic compartments including axon terminals, cell bodies and dendrites [6,7]. Developing neurones in the central nervous system undergo dramatic changes in electrophysiological properties that may, at least in part, be attributable to changes in BK channel function [8]. Increasing evidence suggests that BK channel expression is up regulated during vertebrate central nervous system (CNS) development, dependent on changes in gene transcription, trafficking of channel protein to the plasma membrane as well as posttranslational modification [9-13]. Furthermore, developmental changes in the functional properties of BK channels, including differences in gating behaviour, calcium and voltage sensitivity, as well as regulation by cellular signalling pathways have been reported [14-16]. However, the molecular basis for changes in BK channel phenotype during CNS development is poorly understood.

**Figure 1:**
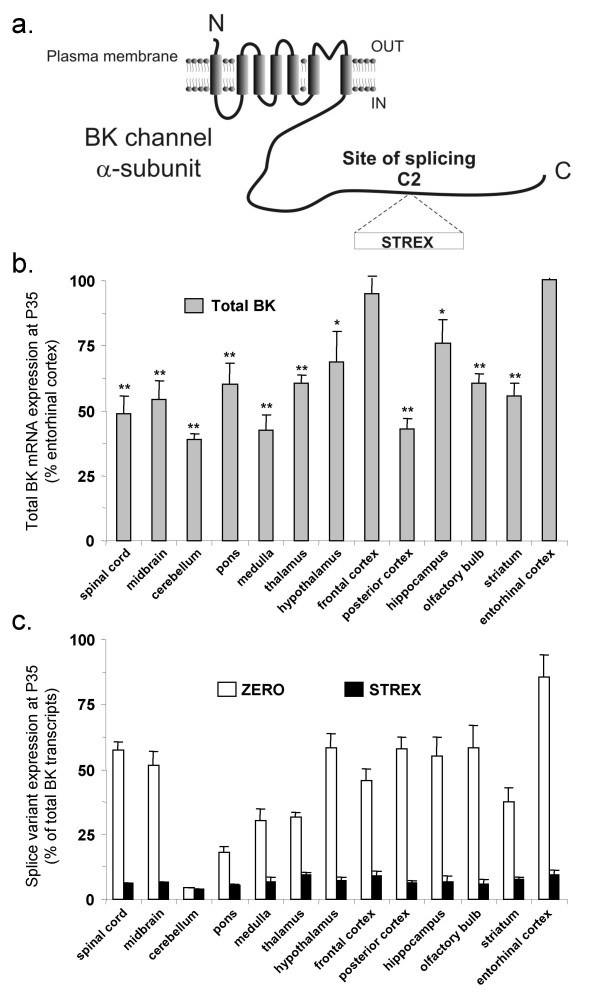
**Total BK channel, and splice variant, mRNA expression in different regions of the murine CNS at postnatal day 35 (P35)**. **a) **Schematic illustrating location of site of splicing C2 and the STREX insert in the intracellular C-terminus of murine BK channel pore-forming α-subunits. The ZERO variant has no insert at site of splicing C2. **b) **Total BK channel mRNA expression (grey bars) in different regions of the CNS from 35-day old (P35) mice. Total BK channel mRNA expression is normalised to β-actin in each region and then displayed as a percentage of the expression in entorhinal cortex. Data are Means ± S.E.M, n = 5/tissue region. * p < 0.05, ** p < 0.01, compared to entorhinal cortex, Kruskal-Wallis non-parametric test with post hoc Dunn's test for multiple comparisons. **c**) Proportion of ZERO (open bars) and STREX (black bars) mRNA transcripts, expressed as a percentage of total BK channel mRNA transcripts, in different CNS regions from P35 mice. All data are Means ± S.E.M, n = 5/tissue region.

The pore-forming α-subunits of BK channels are encoded by a single gene that undergoes extensive alternative pre mRNA splicing [17]. Alternative splicing can dramatically modify the functional properties of BK channels including calcium and voltage sensitivity, cell surface expression and regulation by diverse intracellular signalling pathways. Indeed, changes in BK channel alternative splicing in the developing Xenopus, Drosophila and Aplysia nervous system have been associated with changes in BK channel properties and neuronal phenotype [18-20]. However, whether changes in expression of BK channel splice variants occur during mammalian CNS development is essentially not known. In mammals, splicing of the Stress regulated exon (STREX) is dynamically controlled by cellular excitability as well as circulating stress and sex hormones [21-25]. Further, insertion of this exon results in channels with significant changes in BK channel phenotype, compared to the insertless (ZERO) variant (Figure 1a), when expressed in heterologous systems [26-31]. In this manuscript, we have exploited quantitative real-time RT-PCR Taqman™ analysis of BK channel splice variants [27] to test the hypothesis that alternative splicing of the STREX exon is regulated during development of different regions of the murine CNS.

## Results

### BK channel mRNA expression in the murine CNS

We first examined whether total BK, and splice variant, mRNA expression levels differed across distinct regions of the adult (postnatal day 35, P35) murine CNS. To compare the relative expression of total BK channel mRNA transcripts between different CNS regions of P35 mice all data were normalised to the expression of the housekeeping gene, β-actin, in each region. Total BK channel mRNA expression was variable between different regions with the highest levels observed in frontal cortex and entorhinal cortex (Figure 1b). Regions with the lowest levels (< 50% of the level in entorhinal cortex) were cerebellum, medulla and posterior cortex. The spinal cord, midbrain, pons, thalamus, hypothalamus, hippocampus, olfactory bulb and striatum displayed intermediate total BK mRNA levels (Figure 1b).

Differential expression of the STREX and ZERO BK channel splice variant mRNA was also observed across these tissues at P35. The relative proportion of STREX transcripts (expressed as a percentage of total BK mRNA in each CNS region) was low in all regions tested, with levels typically < 10% of total BK mRNA transcripts. However, even across tissues, the relative proportion of STREX transcripts was significantly different (p < 0.05, Kruskal Wallis test with post-hoc Dunn's test compared to entorhinal cortex) with frontal cortex, thalamus and entorhinal cortex displaying the highest proportion of STREX transcripts and the pons and cerebellum displaying the lowest levels (Figure 1c). The proportion of total BK channel transcripts expressing the ZERO variant also varied significantly across tissues. The entorhinal cortex displayed a significantly (p < 0.01, Kruskal Wallis test with post-hoc Dunn's test) greater proportion of ZERO transcripts compared to any other tissue, with 85.6 ± 9.7 % of total BK transcripts encoded by the ZERO variant. (Figure 1c). In the majority of CNS regions, the proportion of ZERO transcripts was greater than 50% of total BK mRNA transcripts with the notable exception of cerebellum, in which 4.5 ± 2.3 % of transcripts encoded for the ZERO variant (Figure 1c). Taken together, these data suggest that the ZERO variant is the predominant transcript in most adult CNS tissues and that the STREX variant is expressed at a significantly lower level at postnatal day 35. A notable exception is the cerebellum, in which both ZERO and STREX transcripts represent < 10% of total transcripts at P35 suggesting that other site C2 splice variants are predominantly expressed in this region.

### Developmental regulation of BK channel mRNA splicing in the CNS

In order to investigate developmental changes in BK channel mRNA expression in different regions of the murine CNS, BK channel mRNA expression was quantified at embryonic days 13, 15 and 18, and postnatal days 7 and 35. For each tissue, the total BK channel mRNA expression at each developmental time point was expressed as a percentage of that at P35. For each splice variant the expression at each developmental time point was expressed as a percentage of the total BK channel transcripts in each tissue.

#### Tissues from rhombencephalon, mesencephalon and spinal cord

**Figure 2:**
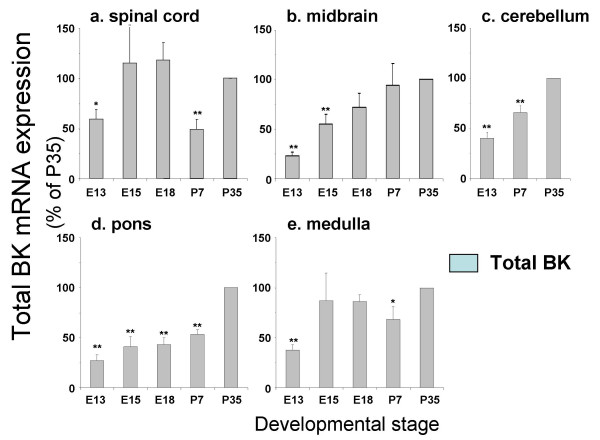
**Developmental regulation of total BK channel mRNA expression in tissues from the rhombencephalon, mesencephalon and spinal cord**. Total BK channel mRNA levels expressed as a percentage of postnatal day 35, in mouse **a) **spinal cord, **b) **midbrain, **c) **cerebellum, **d) **pons and **e) **medulla at embryonic day 13 (E13), 15 (E15), 18 (E18) and postnatal days 7 and 35 (P7 and P35 respectively). All data are Means ± S.E.M, n = 5/tissue region. * p < 0.05, ** p < 0.01, compared to respective P35 data, Kruskal-Wallis non-parametric test with post hoc Dunn's test for multiple comparisons.

**Figure 4:**
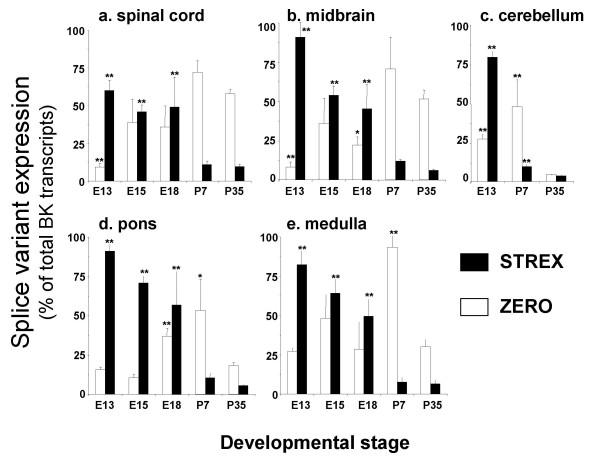
**Developmental regulation of STREX and ZERO variant splicing in tissues from the rhombencephalon, mesencephalon and spinal cord**. STREX (black bars) and ZERO (open bars) mRNA levels expressed as a percentage of total BK channel transcripts in the respective tissue at each developmental time point. Splice variant expression was analysed in mouse: **a) **spinal cord, **b) **midbrain, **c) **cerebellum, **d) **pons and **e) **medulla at embryonic day 13 (E13), 15 (E15), 18 (E18) and postnatal days 7 and 35 (P7 and P35 respectively). All data are Means ± S.E.M, n = 5/tissue region. * p < 0.05, ** p < 0.01, compared to respective splice variant expression at P35, Kruskal-Wallis non-parametric test with post hoc Dunn's test for multiple comparisons.

In spinal cord a small, but significant, increase in total BK channel mRNA expression was observed from E13 compared to P35 (Figure 2a). This was accompanied by a dramatic decrease in STREX variant expression with a concomitant increase in ZERO variant mRNA expression (Figure 4a). Similar developmental up regulation of total BK channel mRNA expression was also observed in the midbrain, cerebellum, pons and medulla (Figure 2b–e). In these tissues, significant developmental downregulation of STREX variant expression was again observed between embryonic and postnatal stages (Figure 4b–e). In midbrain this was paralleled by a significant increase in ZERO variant expression whereas in pons and medulla no significant change between E13 and P35 were observed (Figure 4d &4e). In contrast to other regions, ZERO variant expression significantly decreased with postnatal development in the cerebellum (Figure 4c).

#### Tissues from the Diencephalon and Telencephalon

**Figure 3:**
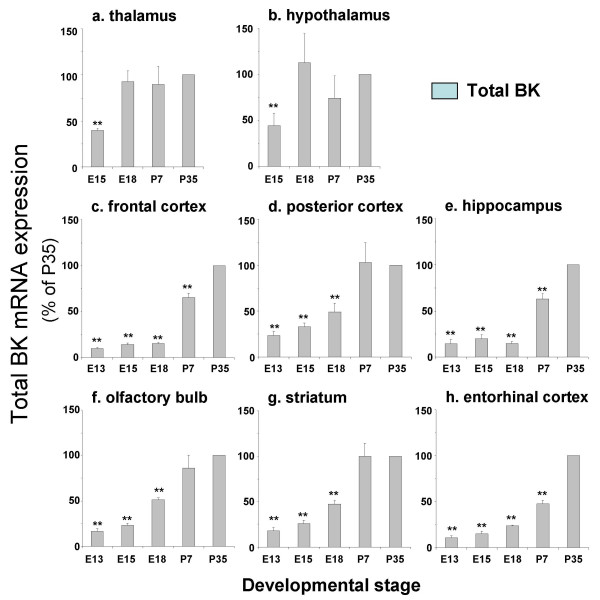
**Developmental regulation of total BK channel mRNA expression in tissues from the diencephalon and telencephalon**. Total BK channel mRNA levels expressed as a percentage of postnatal day 35, in mouse **a) **thalamus, **b) **hypothalamus, **c) **frontal cortex, **d) **posterior cortex, **e) **hippocampus, **f) **olfactory bulb, **g) **striatum and **h) **entorhinal cortex at embryonic day 13 (E13), 15 (E15), 18 (E18) and postnatal days 7 and 35 (P7 and P35 respectively). All data are Means ± S.E.M, n = 5/tissue region. * p < 0.05, ** p < 0.01, compared to respective P35 data, Kruskal-Wallis non-parametric test with post hoc Dunn's test for multiple comparisons.

**Figure 5:**
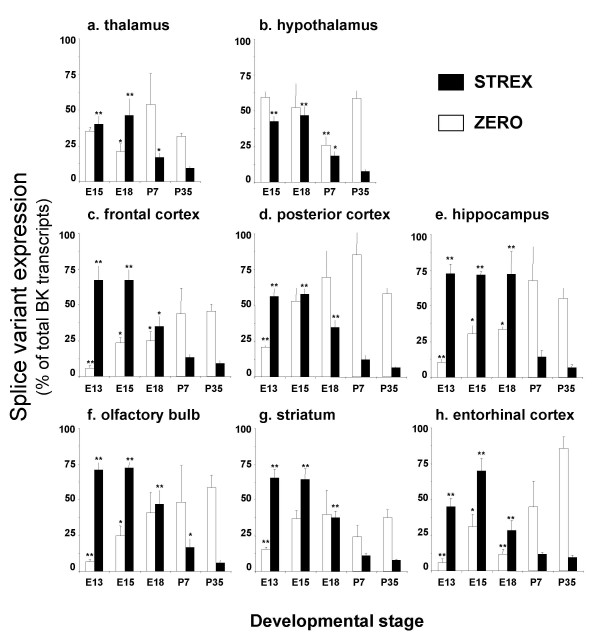
**Developmental regulation of STREX and ZERO variant splicing in tissues from the diencephalon and telencephalon**. STREX (black bars) and ZERO (open bars) mRNA levels expressed as a percentage of total BK channel transcripts in the respective tissue at each developmental time point. Splice variant expression was analysed in mouse: **a) **thalamus, **b) **hypothalamus, **c) **frontal cortex, **d) **posterior cortex, **e) **hippocampus, **f) **olfactory bulb, **g) **striatum and **h) **entorhinal cortex at embryonic day 13 (E13), 15 (E15), 18 (E18) and postnatal days 7 and 35 (P7 and P35 respectively). All data are Means ± S.E.M, n = 5/tissue region. * p < 0.05, ** p < 0.01, compared to respective splice variant expression at P35, Kruskal-Wallis non-parametric test with post hoc Dunn's test for multiple comparisons.

In thalamus and hypothalamus a small, but significant, increase in total BK channel expression was observed from E15 to P35 (Figure 3a &3b). In contrast, total BK channel mRNA expression increased almost 10-fold between embryonic and postnatal stages in frontal cortex, posterior cortex, hippocampus, olfactory bulb, striatum and entorhinal cortex (Figure 3c–h). In all regions examined, there was a significant developmental downregulation of STREX variant mRNA expression (Figure 5). In frontal cortex, posterior cortex, hippocampus, olfactory bulb, striatum and entorhinal cortex this is associated with a significant upregulation of ZERO variant mRNA expression (Figure 5). In thalamus and hypothalamus no significant changes in ZERO variant mRNA expression was observed between E15 and P35 (Figure 5).

## Discussion

The contribution of BK channels to the regulation of CNS function is critically dependent upon cell type, subcellular localisation, intrinsic BK channel kinetic properties, calcium- and voltage sensitivities, and regulation by diverse cellular signalling pathways. Such diversity in the functional properties of BK channels, encoded by a single gene, can be generated by multiple mechanisms including expression and heterotetrameric assembly of distinct splice variants of the pore-forming subunit, association with regulatory beta subunits and signalling complexes and posttranslational regulation. This study suggests that during murine development a contributing factor to the impact of BK channels on CNS function would be through control of alternative splicing of the BK channel pore forming subunit.

The robust developmental changes in splice variant mRNA expression we observe in multiple CNS regions strongly supports the hypothesis that BK channel splicing is coordinated in the developing CNS and is of functional relevance. In all CNS regions examined, the expression of the STREX variant was significantly down regulated in the face of increasing total BK mRNA levels. In most tissues, such as spinal cord and olfactory bulb, this was accompanied by an upregulation in ZERO variant expression suggesting that splicing decisions to exclude the STREX insert are coordinated across all regions of the developing murine CNS. However, there are important exceptions to this rule such as the cerebellum. In the cerebellum, both STREX and ZERO variant expression is developmentally down regulated resulting in ZERO and STREX variants representing < 10% of total BK channel transcripts at P35. In the cerebellum, developmental upregulation of total BK channel mRNA must be accompanied by an increased expression of other site C2 splice inserts. A similar situation must also occur in tissues such as pons and medulla in which STREX expression declines with no significant change in proportion of ZERO variants when comparing between E13 and P35. Analysis of the splicing decisions in CNS regions with distinct splicing patterns should provide important insights into the mechanisms controlling splicing at site C2 during development.

This broad-scale qRT-PCR analysis, at the level of CNS tissue regions, provides a framework in which to generate testable hypothesis of the functional importance of BK channel alternative splicing in specific cell types during murine CNS development. Clearly, detailed expression and functional analysis in individual cell types within a particular brain region are required to address these issues.

In this regard, during the time-span of the developmental regulation analysed here, considerable changes in both maturational states of neurones (including arborisation of dendritic networks, connectivity and intrinsic electrical excitability) as well as the cellular composition of brain regions (e.g. relative levels of glial to neuronal cells) are apparent. Even in the adult state, considerable cell-cell variations in BK channel mRNA expression levels are apparent. For example, in the adult cerebellum total BK channel mRNA and protein levels are high in the Purkinje cell layer but with very low expression in granular cells [3,6,7]. Such cell specific expression levels are also highlighted by both functional and biochemical analysis of BK channel expression in a variety of neurones, including the widely varying levels of BK channel protein observed in detailed histochemical analysis of rat and murine brain [3,6,7]. Thus, whether the changes in splicing decision reported here reflect changes in cellular composition or intrinsic properties of maturing cell types remains to be examined. While these developmental changes in BK channel expression levels are likely to be of functional relevance, it is unlikely that BK channels play a dominant role in proliferation, migration or morphological maturation of the developing CNS as BK channel knockout mice [3] do not show gross abnormalities in neuronal or brain architecture. This suggests that changes in BK channel expression during development are more important for shaping cellular activity, plasticity, and/or connectivity.

A further caveat to these studies is the extent to which developmental changes in BK channel mRNA levels in fact reflect changes in the expression, or functional properties, of BK channel protein. While determination of the functional consequence of BK channel mRNA splicing during development remains to be fully explored in vertebrates [20], the developmental upregulation in total BK mRNA expression is in accordance with several functional and molecular studies [9-13,15,16,20]. For example, in the rat cerebellum, total BK channel mRNA expression increases in the first 2 weeks of postnatal development, a process that appears to be activity dependent [12]. In chick ciliary neurones, developmental upregulation of BK channel mRNA is observed at E8, prior to synaptogenesis, and before a significant macroscopic BK current is observed [9,10,13]. In chick ciliary neurones, the increase in functional BK channel expression is dependent upon target-derived factors [9,10,13]. Finally, the relative expression profiles of total BK channel mRNA levels at late postnatal stages are in broad agreement with the distribution of channel protein in adult mice [6].

What may be the functional consequence of differential splicing of the STREX exon during murine CNS development? The STREX insert represents a gain-of-function module that confers STREX variant channels with faster activation and slower deactivation kinetics and channels are activated at more negative voltages than other variants [23,27,30]. As such, STREX channels have been proposed to support high frequency action potential firing, for example in chromaffin cells [32], although this function is likely to be context and cell type dependent. In humans, gain of function mutations in the BK channel pore-forming subunit result in generalised epilepsy [33]. Furthermore, in mice, genetic ablation of the neuron specific β 4 regulatory subunit results in a gain of function of BK channels resulting in hyperexcitability [34]. Thus, the general down regulation of the gain-of-function STREX variant, from embryonic to postnatal development, may provide a protective mechanism to limit hyperexcitability in the postnatal CNS. In addition, STREX variant channels display distinct posttranslational regulation by a variety of cellular signalling pathways compared to other variants: including differences in regulation by protein phosphorylation, cellular REDOX potential and hypoxia [28,29,31]. Taken together, these attributes may allow neonates to differentially control BK channel function, compared to adults, dependent upon the prevailing physiological demands. In this regard, the close association of BK channels with either voltage- or ligand- dependent Ca^2+ ^entry pathways [35-37] may play an important role in shaping Ca^2+ ^– signalling to the nucleus to programme developmental changes in gene transcription [38]. Clearly detailed biochemical and functional analysis of BK channel splice variant expression is warranted in selected systems to address such issues.

How might alternative splicing of the BK channel be regulating during development? Inclusion of the STREX exon is dynamically regulated by cellular activity *per se *through calcium dependent activation of calmodulin kinase IV [24,25]. Indeed, in murine cerebellar granule cells, STREX variant expression is controlled by calcium entry through L-type calcium channels and genetic deletion of calmodulin kinase IV in the cerebellum results in an upregulation of STREX transcripts compared to wild type controls [25]. Furthermore, both stress and sex hormones potently regulate alternative splicing of the STREX exon in endocrine tissues [21-23] and such hormones have dramatic effects on neuronal development, activity and plasticity [39-41]. Clearly, such multifactorial control of the splicing decision suggests that expression levels of splice variants will be critically dependent upon the cell type under investigation. Indeed, although the STREX insert is highly conserved in vertebrate evolution [27] STREX mRNA expression increases in spinal neurones in Xenopus during early postnatal development [20]. However, as robust downregulation of STREX variant expression occurs across all regions of the CNS our data suggest that splicing decisions to exclude the STREX exon from BK channel transcripts is tightly coordinated in the developing murine CNS.

## Conclusion

We conclude that developmental up regulation of total BK channel mRNA levels in the murine CNS are associated with a developmentally regulated switch in pre mRNA splicing. The downregulation of STREX variant expression paralleled with a general increase in ZERO variant expression suggests that changes in the relative expression levels of these phenotypically distinct channel isoforms might result in changes in the physiological function of BK channels during mammalian brain development.

## Methods

### Total RNA and cDNA preparation for qRT-PCR TaqMan™ analysis

Initial transcript profiling was performed using Origene Rapid-Scan murine brain cDNA arrays. Additional analysis was performed on pooled tissue dissected from C57Bl6 mice of the indicated developmental age. Total RNA was prepared using the QIAgen RNeasy Mini Kit according to the manufacturer's instructions. RNA was treated with RNAse free DNAse and reverse transcription performed in 20 μl reactions containing 1 × reverse transcriptase buffer (QIAgen), 0.5 mM of each dNTP, 1 μM oligo-dT primer or random hexamers (Amersham Pharmacia), 10 U of RNasin (Promega), 4 U of Omniscript reverse transcriptase (QIAgen) and 2 μg of total RNA. Reactions were incubated for 60 min at 37°C, then cDNA products stored at -20°C before TaqMan™ analysis. Control reactions were performed in parallel to exclude contamination from genomic DNA including exclusion of reverse transcriptase or primers from reverse transcriptase reaction.

### qRT-PCR TaqMan™ analysis

Primers and probes for TaqMan™ quantitative real-time polymerase chain reaction (qRT-PCR) assays, specific for each murine site C2 splice variant, were designed with Primer Express v1.2 (Applied Biosystems) as described previously [27]. TaqMan™ probes, labelled at the 5' end with FAM (6-carboxyfluorescein) and at the 3' end with TAMRA (6-carboxytetramethylrhodamine), were synthesized by Applied Biosystems.

**Total BK**:

*BKfwd*: CTCCAATGAAATGTACACAGAATATCTC;

*BKrev*: CTATCATCAGGAGCTTAAGCTTCACA;

*BKprobe*: CCTTCGTGGGTCTGTCCTTCCCTACTGTT.

In addition the murine BK channel Assay-on-Demand set (BK-AoD, Assay ID Mm00516078_m1) from Applied Biosystems was also used. Total BK channel mRNA expression was determined from the mean expression using both the total BK and BK-AoD probe-primer sets.

**ZERO**:

*ZEROfwd*: GCCAAAGAAGTTAAAAGGGCATT

*ZEROrev*: CGGCTGCTCATCTTCAAGC

*ZEROprobe*: TGACGTCACAGATCCCAAAAGAATTAAAAAATGTG

STREX:

*e21fwd*: TTTGATTGCGGACGTTCTGA

*e21rev*: TCTCTCAAGGGTGTCCACGTTAC

*e21probe*: CTGCTCGTGCATGTCAGGCCGT

**β-actin**: The murine β-actin Assay-on-Demand set (β-actin, Assay ID: Mm00607939_s1) was used to determine β-actin transcript levels in CNS regions.

All TaqMan™ assays were linear over 7 orders of magnitude and the efficiency, correlation coefficient (R^2^) and limit of detection for each BK channel mRNA assay, determined from a minimum of 3 independent experiments were: **Total BK**: 1.95, 0.99, 0.2 fg cDNA; **.BK-AoD**: 1.95, 0.99, 0.2 fg cDNA; **ZERO**: 1.91, 0.99, 0.2 fg cDNA; **STREX**: 1.98, 0.99, 0.2 fg cDNA. The efficiency and R^2 ^for the **β-actin **assay was 1.95 and 0.99 respectively. To determine specificity of BK channel variant assays, standard curves were also generated for each variant in the presence of a competing concentration of another variant. In each case, no competition was observed even up to a 100,000 fold excess of competing variant.

All assays were performed using Applied Biosystems universal cycling parameters (2 min hold at 50°C, 10 min hold at 95°C, then 40 × (15s at 95°C and 1 min at 60°C) cycles) on an Applied Biosystems ABI Prism 7000 Sequence Detection System. Reactions (25 μl) were performed in ABI Prism 96-well optical reaction plates. Each reaction contained 1 × ABI real-time PCR master mix (including ROX passive reference dye, 5 mM MgCl_2 _and nucleotides), 50 nM each of the respective forward and reverse primers, and 5 nM of labelled TaqMan™ probe. All data were analysed using ABI Prism 7000 SDS software version 1.0 (Applied Biosystems). Transcript expression was determined from standard curves generated using dilutions of the respective splice variant plasmid DNA.

To confirm our ability to accurately discriminate the proportion of STREX and ZERO splice variant transcripts in a total BK channel transcript population, we undertook experiments using varying amounts of cDNAs encoding the STREX and ZERO variant and analysing mixes using both total and splice variant specific TaqMan™ assays. For example, using a constant amount of STREX input (0.2 pg) with varying amounts of zero cDNA allowed us to analyse each variant as a percentage of total BK input. For three independent experiments using a STREX/total BK ratio of: 1%; 10%; 50%; 90% and 99% the experimentally determined ratios were: 2 ± 3%; 11 ± 2%; 50 ± 3%; 90 ± 3%; 97 ± 2%. For the same predicted ZERO/total ratios, the experimentally determined ratios were: 2 ± 5%; 9 ± 4%; 52 ± 2%; 88 ± 3%; 96 ± 3%. Thus STREX or ZERO splice variant levels were expressed as a percentage of the total BK transcripts.

### Statistical analysis

For comparison of total BK channel mRNA expression in different regions of the CNS, from mice at postnatal day 35 (P35), total BK channel mRNA expression was normalised to β-actin and then expressed as a percentage of that observed in entorhinal cortex. To compare developmental changes in total BK channel mRNA expression the total BK channel mRNA levels at each time point were normalised to that at P35 for each tissue type. Statistical analysis between CNS regions at P35 was performed using the non-parametric Kruskal Wallis test, with post hoc Dunn's test for multiple comparisons between groups. For analysis of splice variant expression at each developmental time point, the fraction of total BK mRNA transcripts that encode each splice variant mRNA was calculated from the variant to total BK channel transcript ratio. Splice variant expression is shown as a percentage of the total BK channel transcript level in each region at the respective developmental time point. Changes in splice variant expression, in individual CNS regions at different developmental time points, was analysed using the non-parametric Kruskal Wallis test, with post hoc Dunn's test for multiple comparisons between groups. All data are expressed as mean ± S.E.M with n independent experiments per group.

## Abbreviations

BK, large conductance voltage- and calcium- activated potassium channel. CNS, central nervous system. RT-PCR, real-time reverse transcription polymerase chain reaction. STREX, stress regulated exon

## Authors' contributions

SHFM & MJS performed the real time qRT-PCR assays, SHFM & MJS analysed the results and prepared the preliminary draft. SHFM, PR, HGK & MJS all helped conceive and design the study and all worked on and approved the final manuscript.
